# Better food safety practices

**DOI:** 10.1002/fsn3.218

**Published:** 2015-03-13

**Figure 1:**
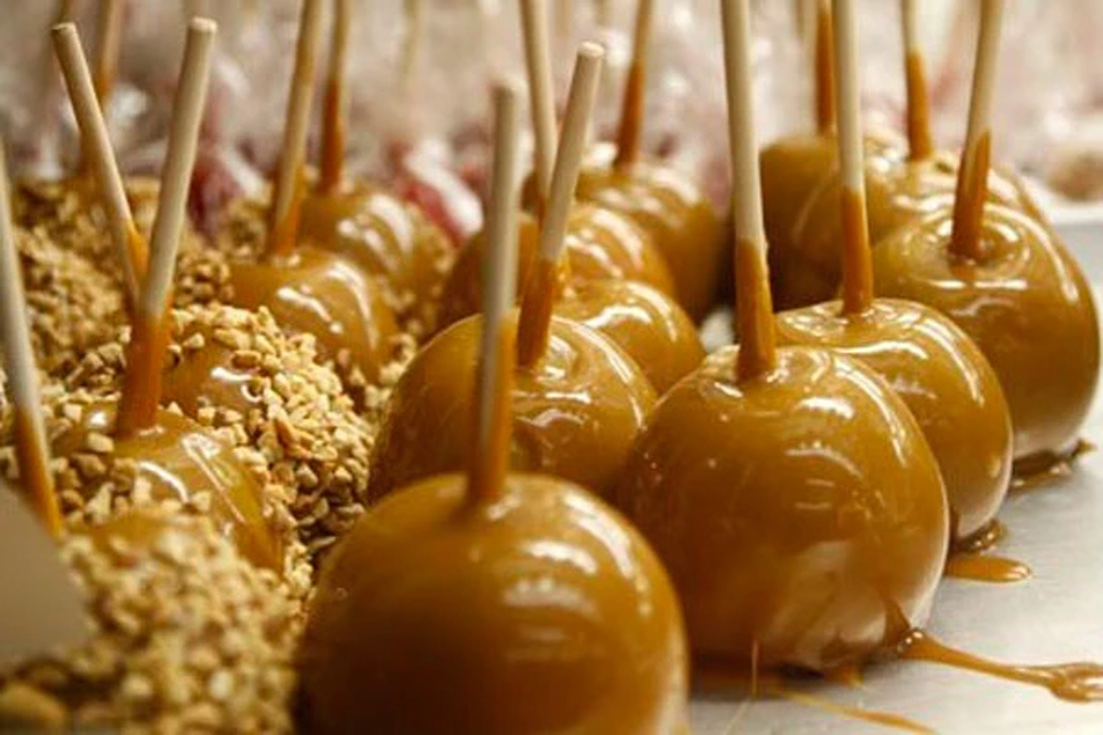
Multistate outbreak of Listeriosis linked to commercially produced, prepackaged caramel apples (CDC 2014).

The fight against foodborne illness is a fight against probability. The US Centers of Disease Control and Prevention (CDC) reported that each year one in six Americans (approximately 48 million) are affected by foodborne illnesses and there are 3000 deaths due to the consumption of tainted food products (CDC 2011). Recent incidences of foodborne outbreaks, including the most recent Listeriosis outbreak linked with prepackaged caramel apples during 2014 Christmas holidays (Fig.1), further confirm the need to detect and identify the often nonconventional sources of contamination as early as possible.

Moreover, the pressure is higher than ever for food manufactures to ensure the safety of food supplies throughout ingredient acquisition, handling, processing, packaging, storage, and distribution. Globally, food safety management continues to move toward “systematic management commitment,” as can be seen both in the US Food Safety Modernization Act (FSMA) as well as the Global Food Safety Initiatives (GFSI) schemes such as Safe Quality Food (SQF), BRC, ISO, etc. The role of effective microbial testing becomes much more critical to the food processing industry.

To date, the majority of small- to medium-sized food processing companies does not have the capability to conduct microbial screening in house. Rather, they rely solely on sending their samples out to commercial testing laboratories, which often give a 4–7 day turnaround time. Not only does such a holding time delay the release of the products to the market, which inevitably shortens the shelf life of the products, but it also requires the processors to allot extra space and pay for extra electricity to store the products on site. Additionally, sending packaged samples to external laboratories for microbial testing also eliminates the opportunity for the food processor to catch where and how the contamination might have occurred, rendering the products susceptible to recurring problems.

Therefore, there is a direct need for the food processing industry to establish on-site microbial screening using tools that are reliable, robust, easy to operate, and cost-effective. Equally, noteworthy is that the food processors need to choose microbial screening protocols and/or instruments that received approval by professional standard-setting organizations such as the AOAC International.

Conventionally, testing every single batch of products is considered cost-prohibitive. With the on-site screening capacity, instead of testing the selected samples of the finished products, more products could be screened in house. Various ingredients, intermittent premixes or semifinished products could also be easily tested for their microbial load before they are employed or processed further. Samples that are tested positive for foodborne pathogens using the in-house screening tool can then be sent out to full-blown microbial laboratories for confirmation or even to conduct challenge studies.

It is widely recognized by the food processing industry that “representative sampling” remains the greatest challenge in managing food safety. The use of ATP (adenosine triphosphate) swap as a sanitation/hygiene indicator has been a widely adopted practice in the food industry's standard sanitation operation procedures (SSOP); however, there remain considerable discrepancies between ATP readings and microbial growth (Wiederoder et al. 2013).

**Figure 2:**
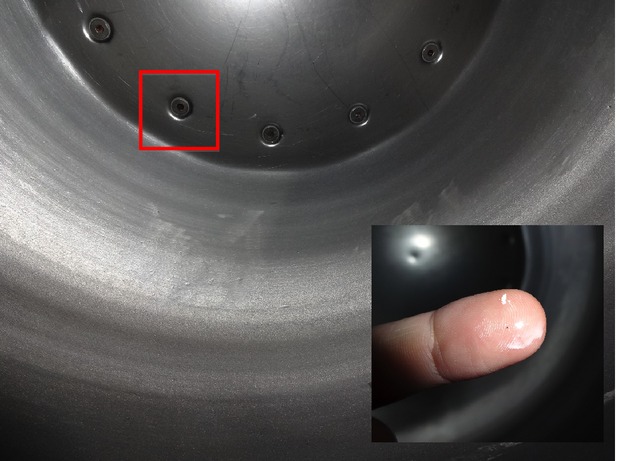
A finger swipe around the screw (red square) on a seemingly clean surface after cleaning and sanitation showed how easily operators could miss critical areas, which could become reservoirs that harbor microbial growth.

**Figure 3:**
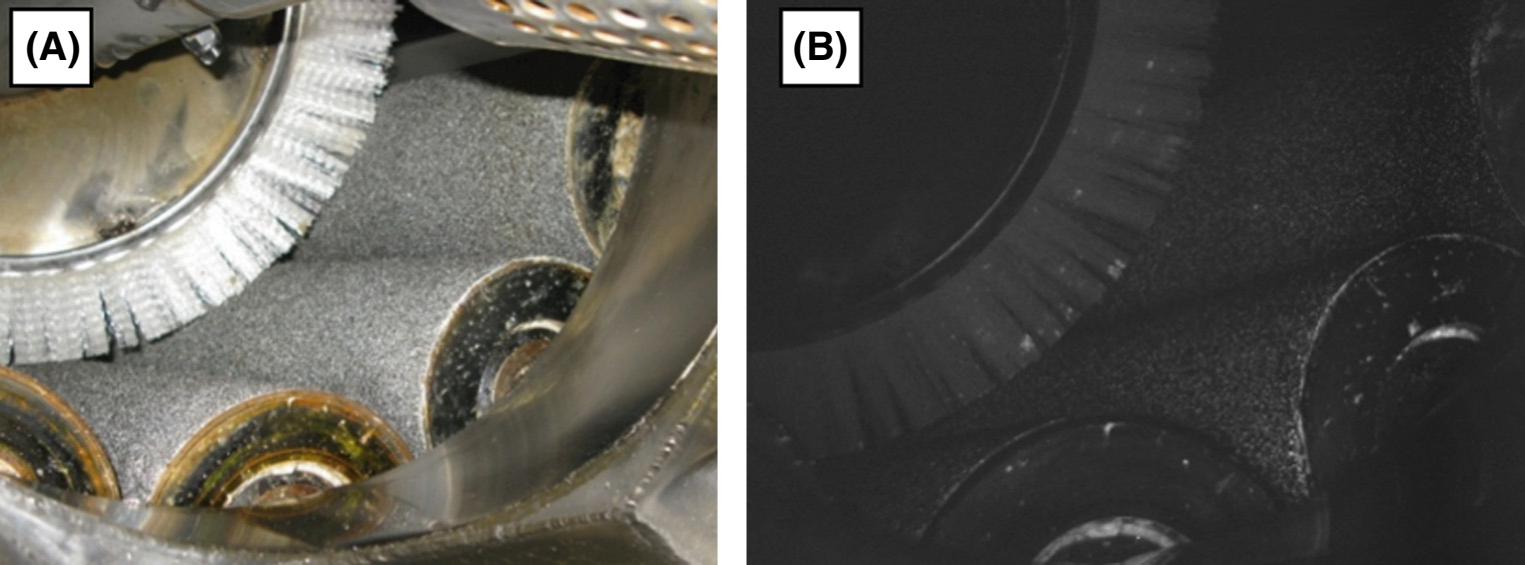
Digital color photo (A) and image taken at 520 nm with UV illumination (B) of a potato peeler showing hidden debris on the brushes after cleaning and sanitation (Wiederoder et al. 2013).

For instance, an unclean surface of processing equipment could easily become a reservoir that fosters growth of pathogenic microorganisms. How can the sanitation supervisor know if the cleaning and sanitation crew have done a good enough job at the end of each cleaning? Where should the QA/QC team sample in order to choose the most representative location for sampling that can reflect the food safety practice in the processing facility? A coating pan that is widely used in the confectionery industry might appear clean upon first glance, but it really requires special attention to critical areas (as seen in Fig.2) to make sure that cleaning is done correctly. In addition to obvious sources of contamination, there are many hidden sources of contamination throughout various parts of processing steps that might not have been realized (Fig.3). Verification of cleaning and sanitation procedures, as well as validation of sampling protocols could be conducted more effectively with in-house microbial screening capacity.

**Figure 4:**
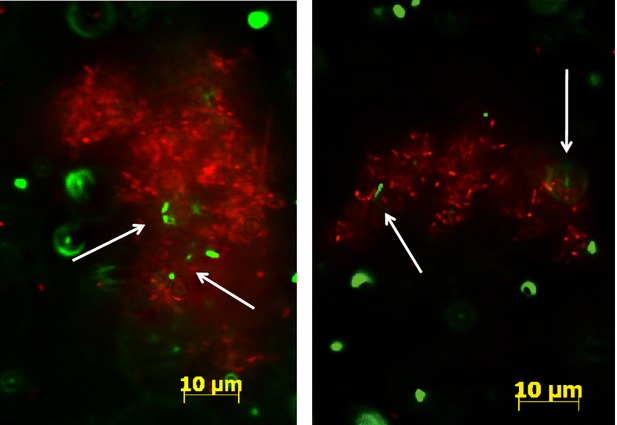
Fluorescence micrographs showing coaggregation between *R. insidiosa* and *E. coli *O157:H7 in fresh-cut processing facilities. Arrows indicated *E. coli* O157:H7 (green) cells embedded in the clusters of *R. insidiosa* (red)(Liu et al. 2013, 2015).

Advancements in microbial biofilm research further confirm the challenges in managing food safety before, during, and after processing. Biofilm formation is a mechanism adapted by many microorganisms that enhances the survival in stressful environments. In food processing facilities, foodborne bacterial pathogens (many of which are poor biofilm formers) could potentially take advantage of this protective mechanism by interacting with other strong biofilm producers (Liu et al. 2013). For example, residential *Ralstonia insidiosa,* a strong biofilm producer frequently isolated from fresh-cut processing environment, have been shown to foster the growth of pathogens (Fig.4) such as *E. coli* O157:H7 (Liu et al. 2015). This once more signifies the need to establish an on-site standby microbial screening tool in food processing facilities.

The most common misconception in the food processing industry has been the concern of bringing pathogenic microbial organisms into processing environment when conducting positive verifications. However, recent advancements in PCR-based detection methods completely eliminate such concerns. By using only a segment of signature DNA representing the target foodborne pathogen such as *E. coli* O157, *Salmonella* or *Listeria*, PCR-based methods could identify existence of pathogenic microorganisms without employing the pathogens themselves as the positive control in the laboratory, hence eliminating the worries of maintaining active culture of pathogens adjacent to food processing areas. In addition, accelerated PCR methods approved by AOAC International have been demonstrated to reduce the detection time to 8–24 h in comparison with typically 2–3 days when using conventional agar plate culture.

Another concern for food industry is the cost associated with setting up an on-site microbial testing laboratory, both in capital and personnel investments. Setting up a food microbiology laboratory typically requires substantial training of laboratory technicians besides investments for plating and media, autoclave and steam generator, subzero and cryogenic freezers for culture storage, incubator, biohood, etc. Contrarily, establishing an on-site microbial screening laboratory only requires a much smaller benchtop space and the PCR-based screening kits are often color coded with foolproof instant computer readouts, making high-throughput handling of samples within reach for the food industry.

It is the editor's intent to stimulate more research and discussions in the area of automated microbial screening of foodborne pathogens in hope to provide accurate, cost-effective, and user-friendly tools that could be readily implemented on-site at food processing facilities. Ultimately, such a heightened screening practice should elevate food safety management for the increasingly interwoven global food processing industry and help the food companies meet FSMA and/or SQF, etc. Not only are researchers encouraged to publish their experimental outcomes via Food Science and Nutrition, but also the food industry is welcome to communicate their practical experiences and/or concerns over existing technologies.
